# What Imaging Technique Correlates Best with Surgical Findings in Gluteus Medius Tendon Tears?

**DOI:** 10.3390/jcm14196714

**Published:** 2025-09-23

**Authors:** Damien Van Quickenborne, Catherine Van Der Straeten, Arne Burssens, Emmanuel Audenaert

**Affiliations:** Orthopaedic Department, Ghent University Hospital, Corneel Heymanslaan 10, 9000 Ghent, Belgium; cathvds@telenet.be (C.V.D.S.); arne.burssens@ugent.be (A.B.); emmanuel.audenaert@ugent.be (E.A.)

**Keywords:** hip, tendon, reconstruction, augmentation

## Abstract

**Background/Objectives:** Gluteal medius tendon tears (GTT) are a common cause of greater trochanteric pain and functional impairment. Accurate preoperative imaging is critical for diagnosis and surgical decision-making. This study aimed to evaluate and compare the diagnostic accuracy of four imaging modalities—X-ray, ultrasound (US), magnetic resonance imaging (MRI), and bone scan (BS)/SPECT/CT—in detecting and grading GTT, using perioperative findings as the reference standard. **Methods:** In this prospective study, a cohort of 45 patients (41 women, 4 men; mean age 62.9) with suspected GTT and failed conservative treatment had open surgical treatment by augmentation of the gluteus medius tendon. All patients underwent preoperative imaging with X-ray, US, MRI, and BS. Imaging results were compared with intraoperative findings. **Results:** MRI demonstrated the highest sensitivity (98%) and strong PPV (91.1%), correctly identifying nearly all true positives. Ultrasound showed similar sensitivity (95%) but yielded more false positives. X-ray and BS exhibited perfect specificity and PPV (100%) but poor sensitivity (21% and 38%, respectively), limiting their utility in ruling out GTT. **Conclusions:** MRI is the most sensitive and reliable single modality for diagnosing GTT, though false positives remain a concern in surgical decision-making. Ultrasound, while sensitive, lacks specificity and should not be used in isolation for surgical decision-making. A multimodal imaging approach, particularly combining MRI with X-ray and BS, may offer high diagnostic certainty and help prevent unnecessary surgical interventions.

## 1. Introduction

Gluteal medius tendon tears are a common musculoskeletal pathology with significant implications for patient mobility and function. Accurate diagnosis and grading of these tears are crucial for determining the appropriate treatment strategy [1].

Various imaging modalities, including radiography (X-ray), ultrasound (US), magnetic resonance imaging (MRI), and bone scans—SPECT/CT (Single Photon Emission Computed Tomography combined with Computed Tomography) (BS), provide valuable diagnostic information, each with distinct advantages and limitations [2,3].

According to recent literature, the best imaging modality for detecting GTT is MRI. MRI has been shown to have high accuracy in diagnosing tears of the gluteus medius and minimus tendons. A study by Cvitanic et al. demonstrated that MRI had an accuracy of 91% for diagnosing these tears, with specific MRI findings such as areas of high signal intensity on T2-weighted images being significantly associated with tendon tears [4].

**Figure 1:**
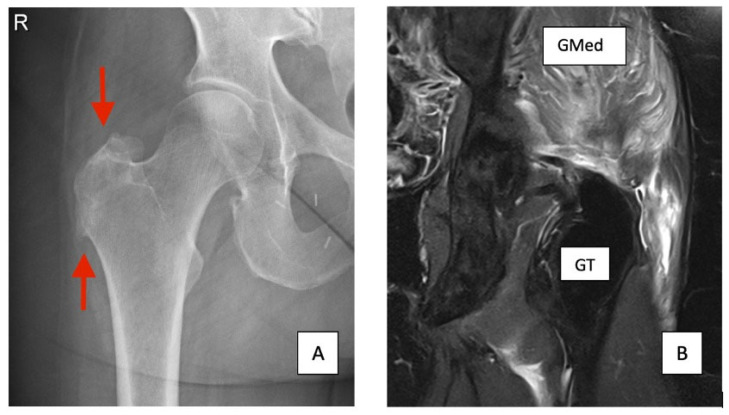
(**A**) Standing AP Xray of the Right Hip with distal spur of the GT and irregular cortex (red arrows) (**B**) T2 MRI Left hip with fatty degeneration of the GMed and liquid filled bursa and discontinued Gluteal tendon.

**Figure 2:**
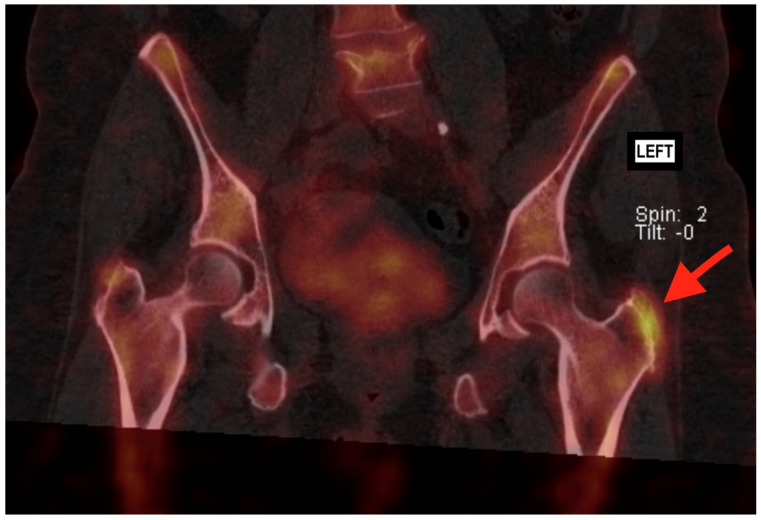
SPECT-CT (Tc-99m bone scintigraphy) imaging of a female patient with a clear hotspot on the left hip (red arrow). Indicating high bone activity in relation to spur formation.

While ultrasound (US) can also be used to identify gluteal tendon pathology, including tendinopathy and tears, it is generally considered less reliable than MRI. Docking et al. found that while US could identify pathological tendons, it had limitations in differentiating between tendinosis and partial-thickness tears [5]. Also, US is more operator dependent than MRI. Therefore, MRI remains the preferred imaging modality in the current literature. X-rays are primarily useful for evaluating bony structures and can help identify associated findings such as calcifications or enthesophytes at the greater trochanter, which may suggest chronic gluteal tendon pathology. However, X-rays do not provide direct visualization of soft tissues like tendons and are not reliable for diagnosing gluteal tendon tears [6]. Sometimes, specific spur formation can be seen on the greater trochanter (Figure 1) [7]. Bone scans, including Tc-99m bone scintigraphy, can detect areas of increased bone turnover, which may be associated with chronic tendon pathology or stress reactions. Very little literature is available on the relationship between bone scans and GTT. A study by Weissman et al. from the American College of Radiology (ACR) highlights that BS with SPECT-CT are useful in detecting various conditions related to hip arthroplasty, but they are not effective in identifying soft tissue abnormalities such as GTT (Figure 2) [8].

The primary objective of this study was to determine the effectiveness of each imaging technique in diagnosing and grading Gluteal Tendon Tears (GTT). By comparing preoperative imaging with intraoperative findings, we aimed to identify the most reliable diagnostic method for guiding clinical decisions and determining the need for surgical intervention in cases of GTT.

## 2. Materials and Methods

### 2.1. Study Design

A total of 45 patients with suspected GTT were analyzed in this prospective observational study, comprising 41 women and 4 men, with an average age of 63 years (range: 36–83 years). Each patient underwent a series of imaging assessments, including X-ray, ultrasound (US), magnetic resonance imaging (MRI), and bone scans (BS). The inclusion criteria consisted of patients presenting with clinical symptoms indicative of gluteal tendon pathology, confirmed through at least two positive imaging modalities. Patients presented with a preoperative score on the VISA- G-Dutch questionnaire of less than 56 (Mean 42.5; Range 27–56) (Table 1).

The VISA-G-Dutch is a validated Dutch version of the Victorian Institute of Sport Assessment–Gluteal questionnaire, designed to evaluate pain and function, specific in patients with greater trochanteric pain syndrome. Scores range from 0 (severe disability) to 100 (full function), with lower scores indicating greater impairment [9].

Patients with a history of prior hip surgery or those diagnosed with autoimmune or connective tissue diseases affecting tendons, such as rheumatoid arthritis, systemic lupus or Marfan syndrome, were excluded.

All patients had previously undergone nonoperative treatment methods, including ultrasound-guided corticosteroid injections and physical therapy, which no longer provided adequate pain relief. Due to the failure of conservative management, surgical intervention aiming at surgical restoration of the GTT, was indicated for the entire cohort, allowing direct visualization and confirmation/scoring of tendon pathology in each case.

Before surgery, ultrasound examination of the greater trochanteric region was performed in all patients by a specialist in the field at our hospital. In 38 patients, we performed a SPECT/CT (BS) to exclude osteoarthritis of the hip. All patients had an MRI and X-ray before surgery. The MRI scans were independently reviewed by two radiologists specialized in musculoskeletal MRI, and their findings were compared to reach a consensus for each patient.

Preoperative imaging results were compared with intraoperative findings to assess the diagnostic accuracy of each modality in identifying GTT. Perioperative evaluation of all 45 patients revealed tears with severity ranging from 1 to 3 on a scale of 0–3. The mean tear severity score was 2.3, indicating a predominance of severe tears in our study population. No patients presented with a score of 0 during perioperative assessment.

The surgical procedures were performed by the same surgical team using a novel surgical technique [7]. Informed consent was obtained from all patients prior to surgery. The minimum postoperative follow up was 12 months. Ethics approval was given by the ethics board of the hospital.

Postoperative management included 6 weeks with two crutches and progressive weight bearing from partial weight bearing (PWB) to full weight bearing (FWB). No strength training or physiological therapy was prescribed during the recovery period.

Preoperative imaging reports were reviewed to assess gluteus medius tendon pathology. Ultrasound and MRI findings were graded on a four-point scale: 0 = normal tendon, 1 = tendinosis without tear, 2 = partial-thickness tear, and 3 = full-thickness tear. X-ray spur formation was scored on a three-point scale: 0 = absent, 1 = mild, and 2 = definite spur. Bone scan (SPECT/CT) findings were scored on a four-point scale: 0 = absent, 1 = mild, 2 = moderate, and 3 = marked uptake. For statistical analysis, patients with a score of 0 or 1 were categorized as negative for gluteus medius tendon tear (GTT), whereas scores of 2 or 3 were considered positive.

This grading approach for US and MRI is descriptive and parallels systems used in the surgical and radiologic literature. The most widely accepted classification distinguishes between normal tendon, tendinosis (no tear), partial-thickness tear, and full-thickness tear [5]. The X-ray spur scoring system is supported by Hartigan et al. [6]. The scoring approach for BS (SPECT/CT) is straightforward and reproducible in daily clinical practice, although it has not been formally validated or described in the existing literature.

Those imaging scores were compared with the intraoperative observations, which were classified as 0 = absent, 1 = tendinitis without tear, 2 = partial tear, and 3 = complete tear. Patients with a score of 0 or 1 were categorized as negative for GTT, 2 or 3 indicated as positive for GTT.

### 2.2. Statistical Analysis

We analyzed the diagnostic performance of four imaging modalities (X-ray, US, MRI, and BS) compared to perioperative findings, using a threshold score of ≥2 to define a positive result for both imaging and perioperative findings.

Descriptive statistics were used to summarize demographic data. Sensitivity and positive predictive values were calculated for ultrasound findings. The same analyses were performed for X-ray, MRI, and BS [10].

## 3. Results

All patients had positive findings on at least two imaging modalities, which served as the indication for surgery. Intraoperative evaluation confirmed gluteus medius tendon tears in 41 cases, while 4 patients showed no tear despite positive preoperative imaging. Perioperative grading confirmed tears in 41/45 patients, with most classified as grade 2 or 3 (mean severity 2.3). MRI grading showed a predominance of grade 3–4 findings, ultrasound most frequently identified grade 2–3, while X-ray spur grading was usually 0–1. Bone scan (SPECT/CT) was performed in 38 patients; in this subgroup, most scans were negative or showed grade 2 uptake consistent with focal activity at the greater trochanter.

### 3.1. X-Ray

X-ray demonstrated limited sensitivity but perfect specificity in detecting lesions. Of the 45 cases evaluated, X-ray correctly identified 9 true positives (TP) and 4 true negatives (TN), while failing to detect 32 lesions (false negatives, FN). No false positives (FP) were observed. This resulted in a positive predictive value (PPV) of 100%, indicating that all lesions identified by X-ray were confirmed perioperatively. However, the negative predictive value (NPV) was notably low at 11.1%, reflecting X-ray’s limited ability to rule out pathology when negative.

### 3.2. Ultrasound

Ultrasound showed excellent sensitivity but limited specificity. Among the 45 cases, ultrasound correctly identified 40 true positives with only 1 false negative. However, 4 false positives were recorded, with no true negatives. The resulting PPV was 90.9%, demonstrating good but not perfect accuracy for positive findings. The NPV was 0%, though this should be interpreted with caution given the small number of negative test results.

### 3.3. MRI

MRI demonstrated the highest overall diagnostic performance, correctly identifying all 41 positive cases (true positives) with no false negatives. Similar to ultrasound, 4 false positives were observed with no true negatives. MRI achieved a PPV of 91.1%, comparable to ultrasound. The theoretical NPV was 0%, though this calculation is limited by the absence of negative test results in our cohort. These findings match the findings of Cvitanic et al. [4].

### 3.4. Bone Scan—SPECT/CT

Similar to X-ray, bone scan showed excellent specificity but limited sensitivity. Among the 38 cases evaluated (not all patients received a BS), BS correctly identified 16 true positives and 4 true negatives, while missing 25 lesions (false negatives). No false positives were recorded. This yielded a PPV of 100%, confirming that all lesions identified by bone scan were verified perioperatively. The NPV was low at 13.8%, indicating limited utility for ruling out pathology.

### 3.5. Comparative Analysis

When comparing the four imaging modalities, MRI demonstrated the highest sensitivity, correctly identifying all positive cases. Both X-ray and bone scan showed perfect specificity (100% PPV) but relatively poor sensitivity, with X-ray missing 32 positive cases and bone scan (SPECT/CT) missing 25. Ultrasound provided a balance of high sensitivity with reasonable specificity, missing only one positive case but producing 4 false positives.

The limitations in negative predictive values across all modalities suggest that negative findings should be interpreted with caution, particularly for X-ray and bone scan. The data indicate that MRI and ultrasound are the most reliable imaging modalities for detection of these lesions, with MRI offering marginally superior performance (Table 2).

### 3.6. Diagnostic Imaging Combinations and Predictive Accuracy

We evaluated pairwise combinations of imaging modalities using an AND logic approach, where a positive result was defined only when both modalities were positive (score ≥ 2). MRI combined with either Bone Scan or X-ray demonstrated a particularly favorable diagnostic profile.

MRI alone showed perfect sensitivity (1.00), detecting all confirmed lesions, but its specificity was limited due to the absence of true negative cases. In contrast, Bone Scan and X-ray each demonstrated perfect specificity (1.00) and PPV (1.00), but low sensitivity. When combined with MRI, these modalities helped filter out potential false positives, significantly increasing diagnostic certainty.

Although this approach reduces sensitivity—since both tests must be positive—it results in higher PPV and specificity. This is beneficial when the clinical priority is to confirm the presence of disease and avoid unnecessary surgical intervention. Therefore, combining MRI with either Bone Scan or X-ray may provide the most dependable preoperative method for identifying clinically significant lesions with high confidence.

## 4. Discussion

This study is the first to evaluated the diagnostic accuracy of four imaging modalities (X-ray, ultrasound, MRI, and bone scan) in the context of GTT, comparing imaging findings with perioperative observations. Our principal findings reveal important strengths and limitations of each imaging modality in clinical practice.

Ultrasound (US), despite being commonly employed in clinical practice for GTT evaluation due to its accessibility, cost-effectiveness, and non-invasive nature, demonstrated diagnostic limitations in our study. While US showed high sensitivity by correctly identifying 40 of 41 true positive cases, it produced 4 false positives (PPV 90.9%). This finding aligns with previous literature suggesting limited evidence supporting ultrasound as a definitive diagnostic tool for GTT [10,11]. The presence of false positives is particularly problematic in surgical decision-making, as it could potentially lead to unnecessary surgical interventions in patients who might benefit from conservative management. Similar challenges with false positive US findings have also been reported by Şirolu et al. [12].

Our findings demonstrate that surgical decisions for GTT should not rely solely on ultrasound due to the risk of false positives. Ultrasound results require cautious interpretation alongside clinical assessment. When ultrasound suggests surgical intervention, confirmation with additional imaging modalities is recommended before proceeding to surgery [11].

MRI demonstrated superior diagnostic performance, correctly identifying all true-positive cases with no false negatives. However, similar to ultrasound, MRI also yielded false positives (PPV 91.1%). This indicates that while MRI offers excellent sensitivity, its specificity for GTT remains somewhat limited. This is in line with the findings of Grimaldi et al. (2025), who reported that imaging abnormalities are common even in asymptomatic individuals, underscoring the importance of careful clinical correlation when interpreting MRI findings [13].

X-ray and BS (SPECT/CT) both showed perfect specificity (PPV 100%) but limited sensitivity, missing substantial numbers of positive cases (32 and 25 false negatives, respectively). These findings indicate that while positive results on these modalities can reliably confirm pathology, negative results should not be used to rule out GTT [3].

Our findings highlight complementary yields of the different modalities: MRI and ultrasound provided excellent sensitivity, while X-ray and SPECT/CT offered perfect specificity. This demonstrates that each modality contributes differently to preoperative evaluation.

This study has several limitations. The perioperative findings in our cohort, with tear severity scores ranging from 1 to 3 (mean 2.3), represent a spectrum concentrated in the moderate to severe range. This distribution may have influenced the diagnostic accuracy results by favoring imaging modalities that are more effective at detecting higher-grade lesions. Additionally, the cohort size is relatively small, although it is comparable to those reported in the recent literature on this specific topic. Finally, the involvement of multiple radiologists in assessing the imaging modalities introduces potential variability in interpretation. Another limitation is that bone scan (SPECT/CT) was not performed in all patients, which may have influenced the comparative accuracy estimates of this modality. Also, it was non-randomized and non-controlled. Nevertheless, all patients underwent standardized imaging and surgical confirmation, which strengthens the internal validity.

## 5. Conclusions

In conclusion, while imaging is essential for guiding surgical intervention in gluteus medius tendon tears (GTT), our findings highlight the risks of relying on a single modality. Ultrasound, despite its high sensitivity, lacks specificity and may lead to overtreatment. MRI, while highly sensitive, showed a notable rate of false positives. To improve diagnostic accuracy and reduce the risk of unnecessary surgery, MRI or US should be combined with a high-specificity modality such as X-ray or bone scan (SPECT/CT).

A multimodal imaging strategy, integrated with careful clinical evaluation, offers the most reliable approach to preoperative decision-making in GTT management.

## Figures and Tables

**Table 1:** Demographic and baseline clinical characteristics.

Sex (F/M)	41 (91.1%)/4
Age (years)	63.0 ± 10.6 (range 36–83)
BMI (kg/m^2^)	26.0 ± 3.3 (range 21.7–36.5)
Affected side (L/R)	19/26
Preoperative VISA-G (0–100)	42.6 ± 7.9 (range 26–56)

**Table 2:** Diagnostic performance of four imaging modalities—X-ray, ultrasound, MRI, and bone scan (SPECT/CT)—in detecting GTT lesions Metrics: true positives (TP), true negatives (TN), false positives (FP), and false negatives (FN), positive predictive value (PPV), and negative predictive value (NPV).

Modality	Cases	TP	TN	FP	FN	Sensitivity	Specificity	PPV	NPV
X-ray	45	9	4	0	32	Limited	Perfect (100%)	100%	11.1%
Ultrasound	45	40	0	4	1	Excellent	Limited (0%)	90.9%	0%
MRI	45	41	0	4	0	Perfect (100%)	Limited (0%)	91.1%	0%
Bone Scan (SPECT/CT)	38	16	4	0	25	Limited	Perfect (100%)	100%	13.8%

## Data Availability

The raw data supporting the conclusions of this article will be made available by the authors on request.
